# Supplementary figures and images for: Non-asymptotic transients away from steady states determine cellular responsiveness to dynamic spatial-temporal signals
Source: PLoS Comput Biol. 2023 Aug 14;19(8):e1011388. doi: 10.1371/journal.pcbi.1011388 (PMC10449117; doi:10.1371/journal.pcbi.1011388)

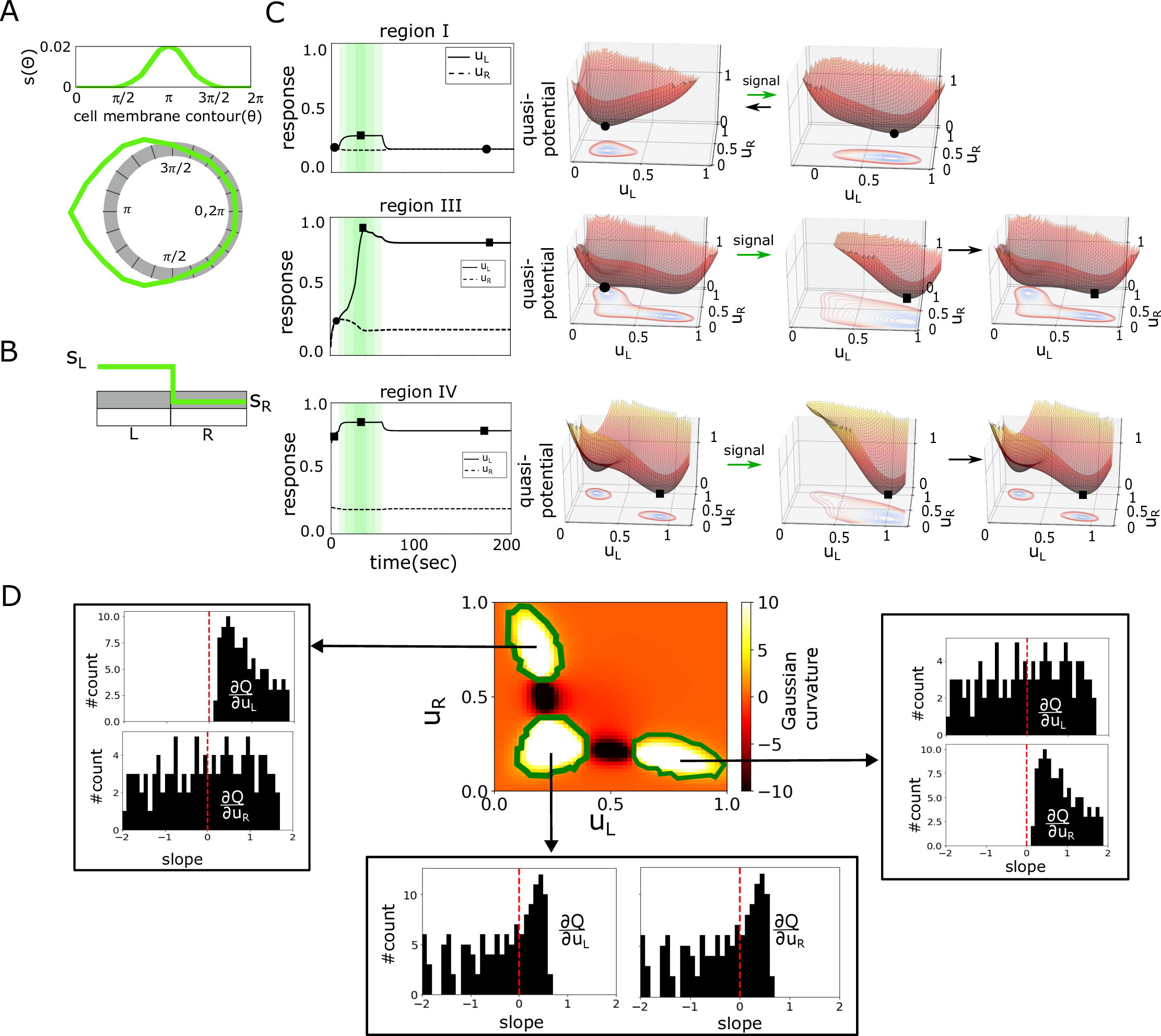

Supplement: S1 Fig — Schematic representation of the gradient signal implementation in (A) the RD simulations, corresponding to Eq (1), and (B) the one-dimensional projection model, corresponding to Eq (3). (C) Time series of u and the quasi-potential landscape transitions during a transient step-like stimulation for organization in region I (top), III (middle) and IV (bottom) corresponding to Fig 1B (same equations and parameters). Green shaded region: signal interval. Circle/square/triangle: non-polar/polar/transient-polar (memory) state. (D) Exemplary estimate of Gaussian curvature (middle) and corresponding slopes distribution in (x, y) = (uL, uR) direction for each of the identified regions. Slopes distribution around 0 in both direction in conjunction with positive curvature uniquely determines a well (stable steady state) in the potential landscape. Description as in Fig 1F. (TIF) [file pcbi.1011388.s001.tif]

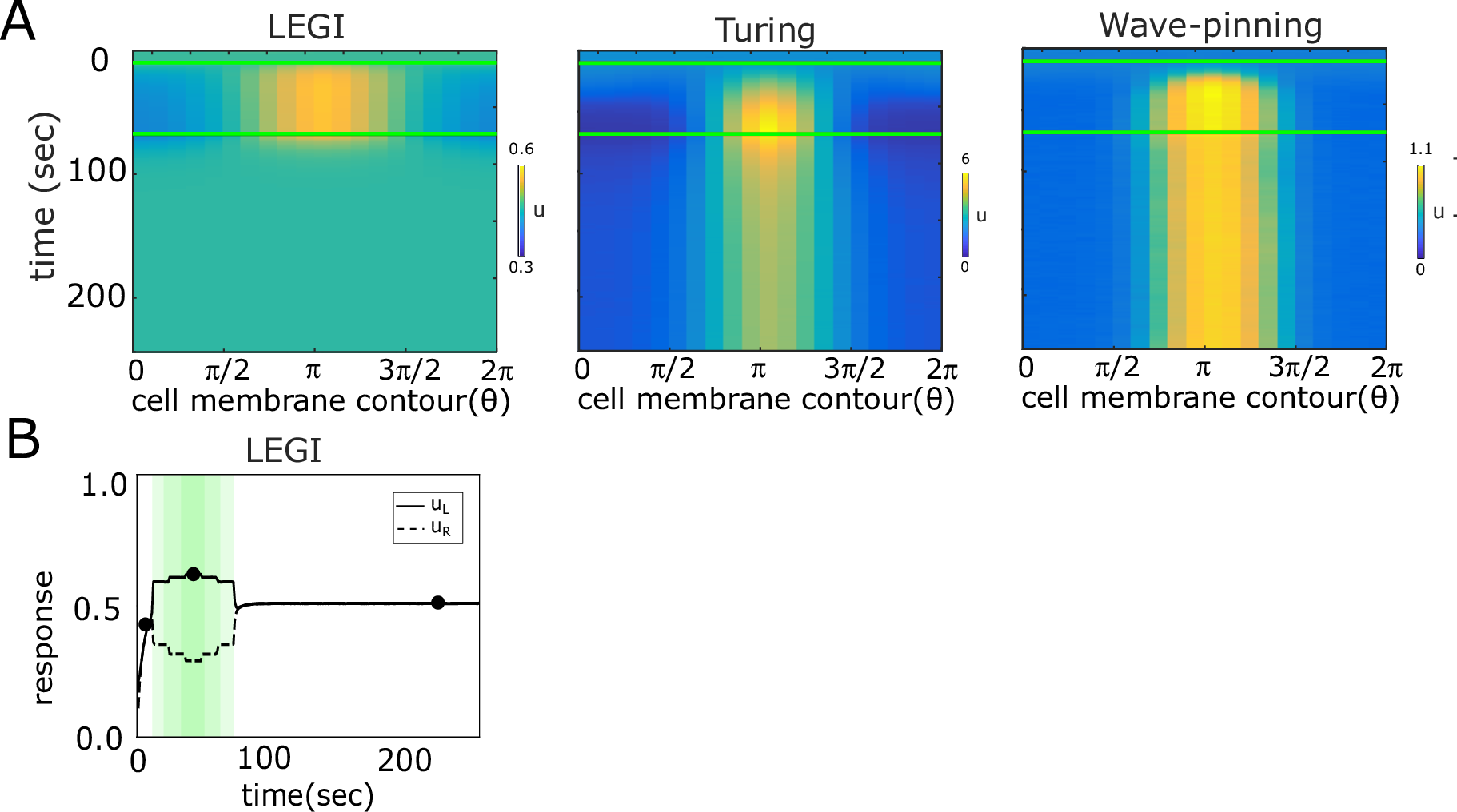

Supplement: S2 Fig — (A) Spatial-temporal response (kymographs) of the membrane-bound active component of the three models. Parameters as in Fig 2, except for Du = Dw = 0.5μm2s−1, Dv = 10μm2s−1 for the LEGI, and Du = 0.1μm2/sec, Dv = 10μm2s−1 for the Turing and Wave-pinning models. (B) Temporal u profile for the LEGI model, corresponding to Fig 2D and 2E. (TIF) [file pcbi.1011388.s002.tif]

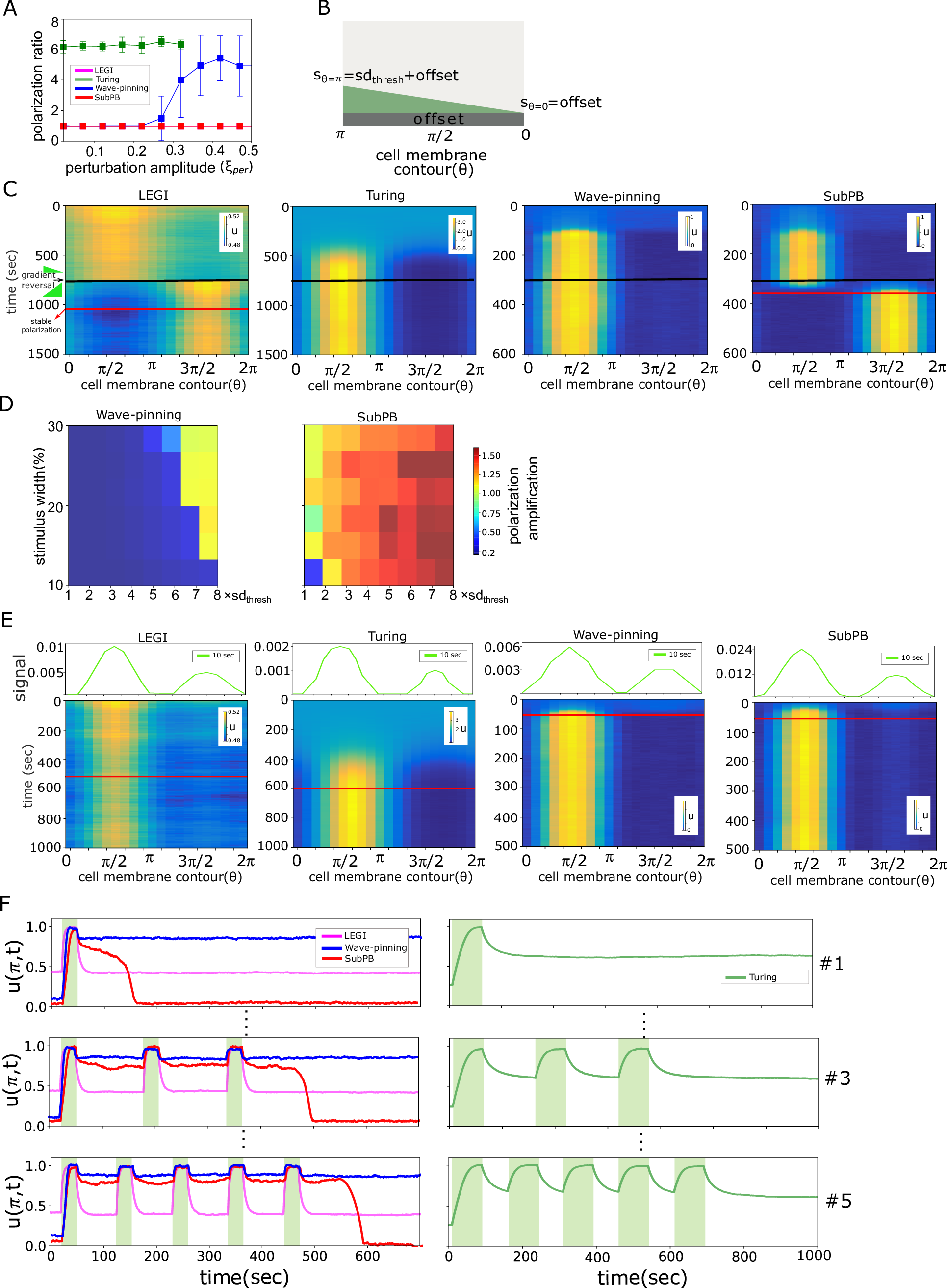

Supplement: S3 Fig — (A) Quantification of spurious activation for increasing perturbation amplitude around the homogeneous steady state. Colors as in Fig 3A. (B) Schematic representation of gradient stimulation with an offset along the cell membrane contour. (C) Kymographs depicting the spatial-temporal response of each of the models to reversal of gradient stimuli (black horizontal line). Red horizontal line: time point when stable reversed polarity is established. (D) Comparison of re-polarization in the Wave-pinning (left) and the SubPB (right) models, for varying stimulus width and maximal stimulus amplitude. (E) Kymographs depicting the spatial-temporal response of each of the models stimulated with simultaneous signals with different amplitudes from opposite cell ends. Red horizontal line: time point where stable polarization with unique axis was established. (F) Exemplary temporal response to consecutive signals from same direction (left: LEGI, Wave-pinning and SubPB; right: Turing model). (TIF) [file pcbi.1011388.s003.tif]
