# Supplementary material for: Non-asymptotic transients away from steady states determine cellular responsiveness to dynamic spatial-temporal signals
Source: PLoS Comput Biol. 2023 Aug 14;19(8):e1011388. doi: 10.1371/journal.pcbi.1011388 (PMC10449117; doi:10.1371/journal.pcbi.1011388)
Supplement: Attachment — Submitted filename: Response_Nandan_etal.pdf [file pcbi.1011388.s004.pdf]

We thank the reviewers for their assessment on our manuscript and the very constructive criticism, insightful and detailed suggestions that we followed to generate the amended version of the manuscript. We are particularly grateful for pointing several references to us which we have unfortunately missed. We have generally restructured the initial part of the manuscript and the description of Figs.1-2 and the corresponding supplementary figures to better explain the SubPB mechanism and its relation and differences to the Wave-pinning model. We have also included additional systematic analysis for the re-polarization (in particular for the Wave-pining model), in light of the references suggested by one of the referees, to further highlight the role of transient dynamics as observed in the SubPB mechanism in responsiveness to dynamic signals. We next provide a detailed response to the comments and concerns raised by the referees.

Reviewer #1: This manuscript concerns generic models of cell polarization in response to imposed spatially non-uniform cues. In particular, it addresses the question of what type of cellular response mechanism can respond to physiological cues in a robust way (definitively and not too sensitively to weak/stochastic cues) while maintaining the ability to adapt to new directional cues.

The authors focus on three broad classes of model that have been proposed in previous publications: local excitation global inhibition (LEGI), Turing-type, and wave-pinning. Using a combination of bifurcation and local stability analysis, and explicit consideration of transient responses to time-varying spatial cues, they illustrate the potential importance of slow transient dynamics in cellular responses. By mapping out the phase space dynamics, they show that LEGI and Turing-type models are not able to account for adaptability, and that the parameter regime typically studied in wave-pinning models also does not account for this. Building on a proposal the authors made for a specific polarization model in a previous publication (Nandan et al., 2022), the authors demonstrate that a different parameter regime for a generic wave-pinning model exhibits criticality close to a saddle-node bifurcation that is associated with slow transient responses to changes in spatial cue. These can result in cellular memory that can underlie a combination of persistence in the face of fluctuating cues with the ability to reorient to new spatial cues.

The general approach adopted by the authors is an important and valuable one, and the focus on transient dynamics in explicitly non-autonomous systems (i.e. those with explicitly time-varying inputs) is quite novel in this context. I agree with the authors that transients and non-autonomy are a neglected aspect of models of cellular dynamics. One reason for this is the challenge of saying anything concrete about possible behaviours in a way that goes beyond simple numerical simulation. An appealing feature of the work reported here is the use of a combination of bifurcation analysis, the construction of pseudo-potential landscapes, and local perturbation analysis to analyse model responses to time-varying cues, focusing on transient behaviours.

We thank the refree for his/hers assessment of the conceptual approach we propose for the study of cellular responsiveness to dynamic signals.

So, I am supportive of the approach used here, and of the message. I think it is an important contribution to understanding the mechanisms of cell polarization and to the necessary move towards exploring transient dynamics of systems in response to time-varying stimuli. However, I think the manuscript needs quite a lot of work to get it in to an acceptable form.

The major problem is that the models are not specified properly and/or clearly -- their functional form and the parameters used. Specifically, there are two principal forms of the models in the manuscript: the spatially continuous form on a circle and the spatially discrete form in two compartments (left and right). It is almost never clear which model is being used. For example, Fig 1C seems to show a simulation of the continuous model (with a Gaussian signal that does not quite correspond to the one stated in Materials and Methods, and with no "spread" or "amplitude" parameters specified), but Fig 1E has an "s_{left}", which presumably refers to some discrete signal in the discrete model (I honestly don't know; as far as I can tell, the meaning of "s_{left}" is never given.)

We thank the referee for pointing to us the lack of clarity regarding the models, and the definitions of the signals. In the amended version of the manuscript we took care that the RD and the 1d projection models are clearly specified when used, we introduce their differences / the purpose, after the description of the model in the main text, and we have included the corresponding references to the equations, as well as the parameters in the figure legends. We have also now defined the shape and implementation of the signals in the RD and the 1d projection model in the main text with more details in Material and methods, and also schematically in Supplementary. Fig. 1A. We hope that this improves the readability of the manuscript.

Specific points:

Eq. (1): Is the way that the signal is introduced (additive on the u equation only) correct? If so, it breaks mass conservation (production of u without any effect on v). Should it also be subtracted from the v equation?

We thank the referee for this comment. Indeed, in order to preserve mass conservation, the signal has been subtracted from the v equation in all the analysis that is presented in the paper. We had chosen not to write this explicitly in the previous version of the manuscript to achieve a simplified representation, however we understand that this might generate confusion in terms of the actual model implementation, and we have therefore amended it in the current version of the manuscript.

Line 60: Perhaps a little pedantic, but f_u and f_v: [0, 2 pi) x R ◊ R. Unless you want to put a modularity condition on the first R.

We thank for the suggestion, however we were unsure at the moment how to properly introduce it in the manuscript and we have therefore left the notation as before, since in our understanding it is not formally wrong. We are however open to modify it.

Line 63: v is defined as a cytosolic component (see also Fig. 1A). However, it is treated in the model as a membrane component (it diffuses in the membrane.) If it was truly cytosolic, it would diffuse "across" the cell, giving a non-local connection in theta. I presume (without reminding myself) that this is the form used (and justified) by Mori et al., but I think it needs a bit of clarification here if v is going to be described as cytosolic.

In the implementation of the model, we followed the description provided by Mori et al. In particular, the diffusion and exchange of the active forms (bound to the cell membrane), and the inactive forms (e.g., RhoGDP in complex with GDIs in the cytosol) are modeled along a cell diameter transect, front to back, approximated as a one-dimensional segment (schematic in Fig. 1A as in Mori et al., 2008).

Line 64: I think mass conservation should be an integral condition (u + v integrated over theta), not just u+v = c_{total} (which doesn't make sense if u and v are dependent on theta.) See above: the current form of (1) breaks mass conservation unless s = 0.

Indeed, mass conservation is an integral condition, and we have re-formulated the description in the manuscript, as the mass conservation holds true for any signal strength.

Fig. 1 legend: 1B introduces regions I-IV. However, these are a bit "orphan" at this stage, as they're not referred to. I think lines 72-106 could be rewritten to make the overall scheme clearer. I suggest that it would be helpful at this stage to explain what the four regions of the wave-pinning model correspond to. As c_{total} increases we have:
I: monostable – only non-polarized HSS exists and is stable;
II: critical – only non-polarizes HSS exists, but there is a ghost of a saddle-node bifurcation;
III: tristable – stable HSS coexists with two stable polarized states (L and R polarized);
IV: bistable – HSS is unstable; two stable polarized states (L and R).
It is then easier to explain the point about criticality: In regions III and IV, a transient signal can/will irreversibly shift the system to one of the stable polarized states; in region II, a transient signal will move the system to a polarised state from which it takes a long time to return to the stable HSS (memory, due to the ghost).
I appreciate that this is in there, but I don't think it is made very clear (and you're not really using the I—IV notation in the text at this stage.) I think you only finally start to make the point clearly in lines 177-193; the points contained here could usefully come earlier.

We thank the referee for this suggestion which we have adopted in the current version of the manuscript. We have therefore re-structured Figures 1 and 2 and the corresponding supplementary figures to:

1) First describe the response to a transient gradient stimulus for the organization in regimes I-IV by means of kymographs (RD simulations) and quasi-potential landscapes description (obtained from the 1d projection model, as described in Materials and methods).

2) We describe that formally region III corresponds to the Wave-pinning and region IV to a classical Turing-like mechanism, whereas region II – SubPB mechanism, and included this in Fig. 1B.
3) We describe in details the features of criticality organization (region II) from aspect of non-autonomous bifurcation analysis and corresponding trajectory description.
4) In Figure 2 we then focus on the LEGI (quasi-potential landscape and trajectory description) and the trajectory description for the Wave-pinning model, and discuss the differences in terms of asymptotic and non-asymptotic dynamics as compared to the SubPB model.

Fig 1D: "response" is not really defined at this point.

As 3 y-labels were required (uL, uR and signal), we have kept response for simplicity, but we have now referred in figure legend that we depict temporal u profile.

Line 85: "critical organization" is not well defined here. See above for a suggestion.

We have taken up the suggestion to describe in more details the regions I-IV as discussed above. We hope that the criticality is now better and more clearly described.

Lines 95-96: It's a matter of semantics, but I think "a step-like approximation approach to the change in the parameter values" is potentially a bit misleading. I would be inclined to describe s(theta, t) as an imposed signal rather than a parameter.

We fully agree that *s* is an imposed signal to the system. However, in order to calculate the subsequent changes in the quasi-potential landscapes, and understand the changes in the bifurcation structure to explain how the non-autonomous dynamics governs the system properties, it is necessary to make the approximation of discreet changes (increase/decrease) in the signal strength. However, following the referee's suggestion, we have now re-formulated the description in order to specify more clearly that *s* is indeed the external signal, and we look at the system's changes for specific signal amplitude values.

Line 96: "discreetly" should be "discretely" (unless you're trying to slip it past the reader without them noticing.)

We thank the referee for pointing this – the statement is re-written.

Lines 107-119: I really don't like the way this little interlude to point out the difference of the critical region to a bistable model disrupts the flow of the analysis of the wave pinning model. I understand the point that the authors want to make, and why they want to make it, but I think it is out of place. If it has to be in, I think it would be much better after line 158. However, I think it would be much more appropriate to use the bistable region (region IV) of the current wave

pinning model to make the comparison, rather than a seemingly ad hoc bistable model (non-mass conservative?) I'd be much happier to see a comparison of transient responses / succession of steady states in the four regions of the main model studied here, rather than the current comparison to a totally different model brought in solely for the purposes of making a point about non-transient responses.

Following the referee's suggestion, we have now excluded the description of the bistable model and rather focus on the description how the system responds for organization in the distinct regions I-IV. Initially, our aim with this model was to show the distinction between IHSS as a global inhomogeneous state, to a local polarization due to signal presence in a model that is characterized by two homogenous steady states. However, we fully agree that this distracts the reader at this point and we have removed this model in the amended version of the manuscript.

Lines 165-167: Please refer to the equations for the LEGI and Turing models here (Eqs. (18) and (19), though the latter need a bit of work, I think -- see below.)

We have amended the reference to the models, and as specified also above, we always aimed to include when RD/ 1d projection models were used in the analysis.

Line 198: I think s_{left} and s_{right} have still not been defined at this point… And, unless I am missing something, (s_{left} - s_{right}) x 100 is not a percentage difference. For that, you're going to need a denominator something like 0.5x(s_{left} + s_{right}).

In the amended version of the manuscript, we describe the functional form of the signals for the 2 model classes (RD and 1d projection models) and made a schematic representation in Supplementary. Fig. 1A. We use s_L, s_R for 1d projection model and $s(\theta, t)$ for RD simulations as they are defined on a circular domain.

Regarding the formulation of (s_L-s_R)X100 as a percentage difference – we fully agree with the referee that doesn't define percentage. We have however adopted this formulation in order to match description that is usually found in experimental manuscripts (e.g. Servant et al., MBoC 10, 1999), where ((s_L-s_R)/s_R)x100 depicts the percentage difference that a cell can sense across its diameter. Although in our analysis s_R is generally kept to 0 (except when there is an offset amplitude), we decided to keep this formulation as the description that could be most closely compared to experimental observations.

However, given the distinction in the current version of the manuscript to use s_L and s_R only for the 1d projection model, we now described it as $(s_{\theta=\pi} - s_{\theta=0}) \times 100$.

Lines 203-204: “the polarization ratio achieved by the LEGI-type model is relatively small (<1)”. Something doesn’t seem right here. The polarization ratio is defined as u_{left}/u_{right}, which must be >1 (unless the system has polarized in a direction opposite to the applied signal.) There seems to be a problem with pd and % in this paragraph.

Indeed, the polarization is ~1 (slightly larger than 1), and we thank the referee for pointing to us this typo that we have missed.

Lines 208, 211-212: the notion of “spurious activation” is raised, but is not defined. If it is being used as a criterion for judging the appropriateness of different models, a bit more work needs to be done to say what it is.

Under spurious activation we have generally referred to activation to a random perturbation, i.e. noise in absence of a signal such as noisy initial conditions. In order to substantiate our claims, we have now quantified the polarization ratio when increasing the perturbation amplitude (ξ_{per}) and the results are shown in Supplementary Fig. 3A. The simulations consider a random perturbation around the homogenous steady state (u_s, v_s) which is implemented as noisy initial conditions as (($u_s + \xi_{per} * r,\ v_s - \xi_{per} * r$), where r is a random number between [0,1]. Average/s.d. from 10 independent realizations is shown. As the simulation show, both the Turing and the Wave-pinning are susceptible to spurious activation (although to different extent), in comparison to the SubPB model that shows robust polarization only in signal presence.

Line 216 et seq.: It is not clear to me what a “gradient with off-set” is.

Under gradient with an off-set we define a gradient where the basal signal value is off-set by a certain amplitude (schematic now included in Supplementary Fig. 3B)

Line 223: “where as” should be “whereas”.

Corrected.

Line 310: The characteristic equation relates to spatially uniform perturbations, and does not result from substituting (4) in (3) as claimed (which would have diffusion terms.)

We thank the referee for pointing this, we have corrected the description.

Lines 314-315: the sentence doesn’t have an ending.

Corrected.

Eq. (7): It would be better to put the variables in in the RHS (i.e. G_1(u_{left},…)).
Also, this may be personal preference, but I think it would all be a lot more readable if u_{left}

was replaced with u_L, etc. The "= G_1" etc. should be something like ":= G_1" to make it clear that this is being taken as a definition of G_1 (rather than an unspecified identification of two things, one of which is not defined.)

We have now put the variables in the RHS, and amended throughout the manuscript the variables as u_L, u_R etc. However, we opted to keep the notation G_1-G_4 for clarity.

Line 338: The G_i are defined as "reaction terms", but they encode both reaction and diffusion in the RD equations. Conventionally, "reaction term" is used only for the diffusion-independent part. It also feels a bit late after Eq. (7) to say what the G_i are…

We thank the referee for the comment and we have amended this.

Lines 340-341: I don't understand what the authors mean by evaluating the determinant of the Jacobian for the odd mode of perturbation. The Jacobian is evaluated at U_S; it does not depend on the mode of perturbation (it is a local property of U_S.)

Eq (9): It is not clear what F_(lambda) is; it is not defined (I think this is a typo, or I'm missing something.) Also the footwork to get from (8) to (9) is a bit opaque.

In the current version of the manuscript, we have combined the general with the model-specific analytical description of the subcritical PB into one section for brevity, and we have expanded the description on the two points raised by the referee here. In brief, for a given PDE system, the occurrence of zero-crossing eigenvalues leads to either pitchfork or saddle-node bifurcations. The existence of the PB bifurcation is related to the odd mode of the perturbation ($\delta u_L = -\delta u_R$ and $\delta v_L = -\delta v_R$), due to the symmetry associated with this bifurcation. This condition, together with the limit $\lambda \to 0$, defines the existence of a pitchfork bifurcation (Paquin-Lefebvre et al., J. Theo. Biol., 497:110242, 2020).

We have illustrated this with the one dimensional projection of the SubPB model (Eqs. (3) in manuscript), where we have derived analytically the existence of the PB. Here, we would like to additionally add numerical estimation of the determinant of F_λ ($|F\_\lambda|$, Eq. (9) in the current version of the manuscript) for varying values of the bifurcation parameter C_{total} (Fig. 1 bottom), to make this point clearer. The partial derivatives in F_λ are evaluated along the HSS branch (solid and dashed black lines in Fig. 1 top). The bell-shaped curve of $|F\_\lambda|$ shows two zero crossing points that directly correspond to the C_{total} for which PB is observed (compare the bifurcation diagram in Fig. 1B in the manuscript with Fig. 1 in this response).

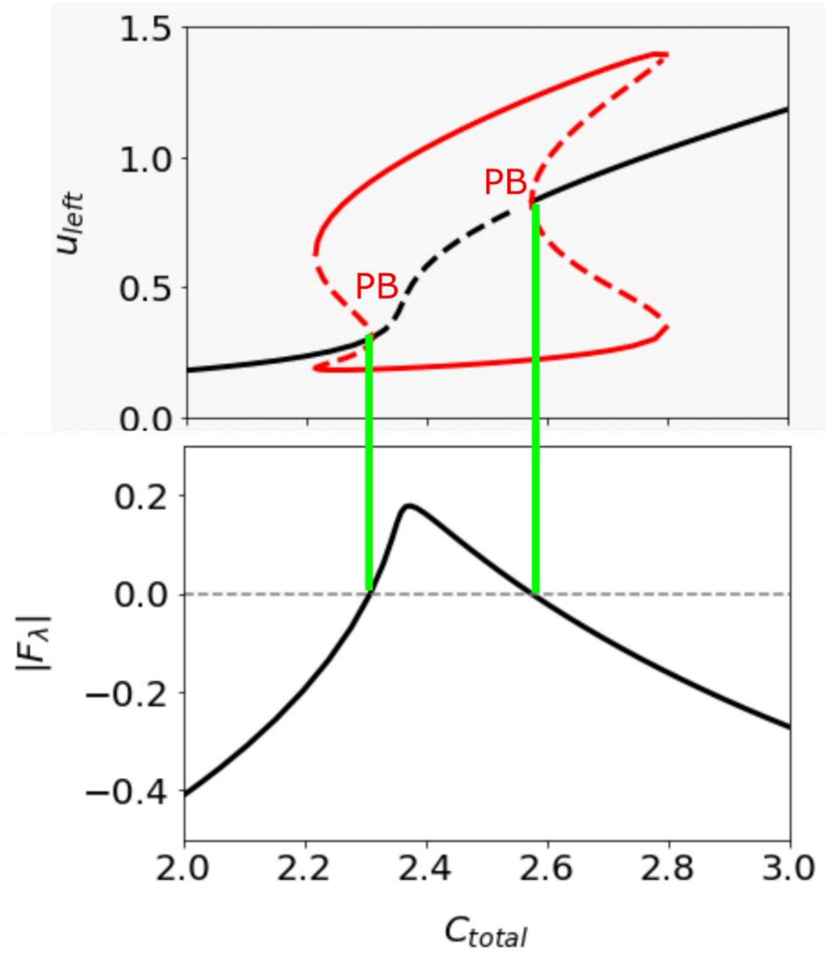

Figure 1: Identification of *PB* using odd mode of perturbation. Top: Bifurcation diagram showing subcritical pitchfork bifurcation same as in Fig.1B in the amended version of the text. Bottom: Determinant of the matrix F_λ (Eq.9 in the amended version of the text) as a function of bifurcation parameter C_{total}. Green lines: marks the zero crossing.

Lines 330-342 (Example): I can't see what role this tangent into an example is playing, and it disrupts the flow of the general analysis. I find it really strange how it ends abruptly with Eq. (9), which doesn't appear to then go anywhere (or tell us anything in particular.) What is Eq. (9) doing in the narrative? If it were up to me, I would cut this tangent completely, or defer it until after the completion of the general analysis (Eq. (15)) and revise it so it actually adds something.

As described to the previous point, we have combined the general with the model-specific analytical description of the subcritical PB into one section, and show analytically the existence of the sub-critical PB for the 1d projection model – equivalent to the numerical bifurcation analysis in Fig. 1B. In the current version of the manuscript, we have expanded the description of analytical steps to make the analysis clear.

Line 349: It should be f_u(u,v) = (k_0 + gamma u^2 /K^2)v – delta u. There is a K^2 missing.

We thank the referee for pointing this mistake on our side which we have amended.

Lines 360-369: It is stated that "Given that D_v >> D_u…". Why is this "given"? Is it given for all models studied? Is it an assumption (required to do LPA), or must it be true for all models of polarization? This section is a bit too vague to be of much help to a reader who hasn't read Holmes et al. It would be better either to just leave a reference to Holmes et al / Grieneisen or to expand it so that it actually gives more useful information for the reader. Either would be appropriate, but as it stands it doesn't really help.

The condition that the two species in the model under study have diffusivities that different by order of magnitude is a requirement to be able to apply the LPA analysis to a system under study. In particular, if $D_v \gg D_u$, it is possible to consider the limit $D_u \rightarrow 0$, $D_v \rightarrow \infty$, and probe the stability of the system with respect to a local perturbation in the form of a narrow peak of the slow variable with a negligible total mass. These are the basic assumptions of the LPA method. In the current version of the manuscript, we have extended the description of the LPA method to improve the clarity.

Lines 373-374: The values for c_{total} for wave-pinning and SubPB are very close (2.25 vs 2.21 – less than 2% difference.) Does it really make sense to claim that cells could accurately "position themselves" so accurately (to take advantage of the saddle node ghost in the way described in this study?) What are the values for D_u and D_v?

We thank the referee for this comment, and a necessary discussion point on the importance of the critical organization. In the abstract models that we use to demonstrate these points, clearly the parameter ranges where the critical organization and thus, the SubPB mechanism or the Wave-pinning mechanism are relatively small, which might imply a fine-tuning mechanism to be required under physiological conditions. However, from the dynamical properties of a ghost of a SN, it is known that the properties are observed in a larger interval before the bifurcation point, having a classical scaling law that the trapping time in the ghost, $\tau \sim 1/(p - p_c)^{1/2}$, where p_c is the parameter value for which the SN_{PB} is observed (Strogatz et al., 2009; Stanoev et al., 2020, Nandan et al., 2022). Moreover, we have demonstrated in our previous experimental study using the EGFR model that ~65% of the single cells investigated showed signatures of critical organization, and quantification of the receptor concentration values on the membrane (which is the bifurcation parameter of the system) showed that the features are observed in a broader interval range. However, whether the system is dynamically maintained at this critical point and if so, what could be the molecular mechanism, remains an open point. We have also observed experimentally that ~25% cells were pre-polarized, which corresponded also to higher receptor concentration on the membrane, that directly follows the general bifurcation structure of the system.

Lines 376-379: The Turing model is not well defined. What is f_v? Is it just -f_u (mass conservation)? If so, say so. What are the values for D_u and D_v?

We have corrected this in the current version of the manuscript.

Lines 390-392: A few questions: 1. Is it right to add the signal term s only to the u equation?; 2. I think the last term in the expression for f_u should be b_1 u v , not g_1 u v; 3. Unlike the other models, this one appears to not be mass-conserving (unless I'm missing something.) Is that right? If so, it would seem to make an even stronger case for using the bistable region of the wave pinning model (region IV) as a comparator rather than this seemingly ad-hoc bistable model; 4. The signal is not properly defined. It just says what the maximum amplitude of signal is without specifying its profile in space and time.)

We respond to the comments raised by the referee:

1. As discussed in the response to the first point by the referee, indeed s figures in the v equation as well, which is not explicitly stated throughout the manuscript.

2/3/4. In the current version of the manuscript, we have removed the description of the bistable model, flowing the referee's suggestion that it breaks the flow of the text.

Lines 415-422:
1. "One dimensional domain of length L = 2 pi R with R = 2" doesn't seem to fit with the rest of the manuscript. For a start, all parameters are dimensional, so R would have to be. But more importantly, the models are posed on the circle, with angular variable theta in [0, 2 pi). This needs sorting out;

The value of cell radius $R = 2\ \mu m$ (unit is amended in the main text) and the length of one-dimensional domain ($L = 2\pi R$) were set the same for all RD simulations performed in the study. However, the x axis of the kymographs that shows the results from these simulations have angular variable (θ) along the cell perimeter instead of actual distance in μm. The scheme in Fig. 1A (in the main text) of the cell with natural periodic boundary condition motivated for such a notation and hence represents the circular symmetry associated with the cell membrane.

2. This section states, almost as an aside, that the ODEs were converted to SDEs with "noise intensity" sigma. A bit more detail is needed here (I'm meant to be able to reproduce the results, and there is no unique way of going from an ODE to an SDE.);

We thank the referee for this comment and we provide an explanation here, as well as an extended explanation in the Materials and Methods section in the manuscript. Generally, this refers to the numerical method for solving the PDEs. The PDE solving method that we have used is as follows. Given a generic reaction diffusion system on a 1D domain,

$$\frac{\partial u(\theta,t)}{\partial t} = f_u(u,v) + D_u \frac{\partial^2 u}{\partial^2 \theta}$$

$$\frac{\partial v(\theta,t)}{\partial t} = f_v(u,v) + D_v \frac{\partial^2 v}{\partial^2 \theta}$$

where $\theta \in [\theta_{min}\ , \theta_{max}]$, the domain is first discretized to N spatial bins,

$$\theta_1 = \theta_{min} < \theta_2 < \cdots < \theta_{N-1} < \theta_N = \theta_{max}$$

with uniform bin size $\delta\theta = \theta_{i+1} - \theta_i$ for $i = 1,2,\ldots,N-1$. The discretized version of the PDE then becomes,

$$\frac{\partial u_i}{\partial t} = f_u(u_i, v_i) + D_u \frac{\partial^2 u_i}{\partial^2 \theta}$$

$$\frac{\partial v_i}{\partial t} = f_v(u_i, v_i) + D_v \frac{\partial^2 v_i}{\partial^2 \theta}$$

where $u_i = u(\theta_i, t)$ and $v_i = v(\theta_i, t)$. Conversion of this PDE to ODE is then done using the *method of lines* (Schiesser, 1991), where the second order partial derivative terms are approximated using finite difference method. This enables us to rewrite the equations with partial derivatives in t as total derivatives,

$$\frac{du_i}{dt} = f_u(u_i, v_i) + \frac{D_u}{\delta\theta^2}(u_{i+1} - 2u_i + u_{i-1}) + O(\delta\theta^2)$$

$$\frac{dv_i}{dt} = f_v(u_i, v_i) + \frac{D_v}{\delta\theta^2}(v_{i+1} - 2v_i + v_{i-1}) + O(\delta\theta^2)$$

Depending on the type of boundary condition, equations at the boundary bins $i = 1$ and $i = N$ are fixed. For example, for periodic boundary conditions two fictitious bins θ_{-1} and θ_{N+1} with constrains $\theta_{-1} = \theta_N$ and $\theta_{N+1} = \theta_1$ are considered. Thus equations at the boundary becomes,

$$\frac{du_1}{dt} = f_u(u_1, v_1) + \frac{D_u}{\delta\theta^2}(u_2 - 2u_1 + u_N) + O(\delta\theta^2)$$

$$\frac{dv_1}{dt} = f_v(u_1, v_1) + \frac{D_v}{\delta\theta^2}(v_2 - 2v_1 + v_N) + O(\delta\theta^2)$$

and

$$\frac{du_N}{dt} = f_u(u_N, v_N) + \frac{D_u}{\delta\theta^2}(u_{N+1} - 2u_N + u_1) + O(\delta\theta^2)$$

$$\frac{dv_N}{dt} = f_v(u_N, v_N) + \frac{D_v}{\delta\theta^2}(v_{N+1} - 2v_N + v_1) + O(\delta\theta^2)$$

This set of equations can now be solved using any standard numerical solver for ODEs. In order to ensure numerical stability of solution we have used explicit Runge-Kutta method of order 5(4) with adaptive time step dt (implemented using *solve_ivp* package in *python*). Truncation error of the order $\sim O(dt^6)$ was sufficient to capture sharp transitions in the reaction diffusion system with spatial discretization with error of the order $\sim O(\delta\theta^2)$.

The external perturbations into the system of ODEs is modeled as a Wiener process where Gaussian white noise is introduced as an additive term at each time step. This results in a stochastic differential equation (SDE) in the Ito form.

$$du_i = \left[f_u(u_i, v_i) + \frac{D_u}{\delta\theta^2}(u_{i+1} - 2u_i + u_{i-1}) \right] dt + \sigma dW(0,1)$$

$$dv_i = \left[f_v(u_i, v_i) + \frac{D_v}{\delta\theta^2}(v_{i+1} - 2v_i + v_{i-1}) \right] dt + \sigma dW(0,1)$$

where $dW(0,1)$ is the gaussian white noise term with unit variance and σ is the noise intensity. Euler-Maruyama algorithm (implemented using *sdeint* package in *python*) was then used to solve this system.

3. "initial conditions are chosen to be around the homogeneous steady steady state" is a bit too vague. The intial conditions need to be stated more concretely than "around".

We have now specified the initial conditions in the description of the spurious activation quantification (Materials and methods).

4. "The stimulus gradient was implemented using a Gaussian function of the form s(theta) = ...". Two points here: first, sigma is used again, when it has just been used for noise intensity in the SDEs. Use two different symbols. More importantly, it is not clear that this form of s(theta) is actually being used in the simulations. For a start, it seems signal is centred on theta = pi, to give a two-sided Gaussian; the form stated would be a one-sided Gaussian with a discontinuity at theta = 0 / 2pi. But actually I can't find any instance where I am certain a Gaussian signal is being used in simulations – it seems to always be a piecewise constant signal (most commonly s_{left} and s_{right}.) This is all very confusing. It is necessary to be explicit and clear about what signal is being applied, for what time duration (beyond just putting green lines on figures) in each numerical simulation. As it is, the reader is left guessing / trying to infer what signal was applied from the results… If the Gaussian form is used in simulations, I couldn't spot any indication of what value of sigma (in this sense) was used.

We thank the referee for pointing out repeated symbols. Now in the amended text, σ represents noise intensity and the variance of the Gaussian signal is represented with w (Materials and methods).

In order to clarify the shape of gaussian signal along the cell perimeter, we added a scheme in Supplementary Fig. 1A (in the amended text) and elaborated the implementation of Gaussian function in a periodic window (see Materials and methods). In brief, for RD simulations, the stimulus gradient was generated using *gaussian* function from *scipy.signal.windows* in Python. This package truncates the gaussian function which otherwise extends from $-\infty$ to $+\infty$ within a given window. For a window of length N (in our case N=20), Gaussian profile is constructed using the expression $s(n) = s_0\, e^{-\frac{1}{2}\left(\frac{n}{w}\right)^2}$ where $n \in [-\frac{N-1}{2} : \frac{N-1}{2}]$, s_0 is the signal amplitude, and

the variance is $w = \frac{(N-1)}{2\alpha}$, varying the value of constant α results in Gaussian profile of varying spread. The generated Gaussian will be maximum at the center and when overlayed on the membrane (Supplementary Fig. 1A top, bottom) results in a maximum at $\theta = \pi$ with negligible discontinuity at $\theta = 0{,}2\pi$. For simulation of the one-dimensional projection models, a step like signal function was used (Supplementary Fig. 1B), with signal amplitude s_L and s_R (generally set to 0). Parameters associated with Gaussian signal are now added in Materials and methods.

Some problems with the references:

The reference "Veronica, G. (2009)…" should be "Grieneisen, V. (2009)…"

Jilkine, A., & Edelstein-Keshet, L. (2011). A comparison of mathematical models for polarization of single eukaryotic cells in response to guided cues. PLoS computational biology, 7, e1001121.

Levine, H., Kessler, D. A., & Rappel, W. J. (2006). Directional sensing in eukaryotic chemotaxis: a balanced inactivation model. Proceedings of the National Academy of Sciences, 103, 9761-9766.

Otsuji, M., Ishihara, S., Co, C., Kaibuchi, K., Mochizuki, A., & Kuroda, S. (2007). A mass conserved reaction–diffusion system captures properties of cell polarity. PLoS computational biology, 3, e108.

Verd, B., Crombach, A., & Jaeger, J. (2014). Classification of transient behaviours in a time-dependent toggle switch model. BMC systems biology, 8, 1-19.

We thank the referee for pointing to us this problems with the references, which are corrected in the amended version of the manuscript.

A plea: Thank you for including line numbers, but the small font size and single spacing made this manuscript really, really hard to read carefully. Please consider submitting with at least 1.5 line spacing in future. It is specified as a submission requirement for a reason.

The 1.5 line spacing is now amended.

Reviewer #2: I found the text very difficult to read, and perhaps because of that I misunderstood what is it that the authors really did.

We thank the referee for his/her comments that we address as follows.

If I understood correctly, they are exploring the well-known wave-pinning model in the presence of the signal that changes in time. They both analytically and numerically investigate the transient polarized cell states in this model discuss important concepts of memory and responsiveness of the cell. I sense that the paper is valuable, however 3 major revisions have to be made:

We would like to point out that there are major difference between the Wave-pinning and the SubPB mechanism and we have further elaborated on these distinctions in the current version of this manuscript. Although both models have as a dynamical mechanism a subcritical pitchfork bifurcation, the Wave-pinning model is organized in a regime where in the absence of a signal, the non-polar and the polarized state coexist, whereas the SubPB model has parameter organization before the SN of the PB, or in a region where in absence of a signal only the non-polar state is stable. Due to this organization at criticality – in the vicinity of the SN_{PB}, the SubPB mechanism gives rise to optimal polarization and signal responsiveness features, as demonstrated in the manuscript.

In the amended version of the manuscript, we have included this in the Fig. 1B, but also re-structured Fig.1 and 2 and the corresponding supplementary figures to explain more clearly the importance of the organization at criticality (the SubPB mechanism) for responsiveness to signals that change over time and space.

1) drastically rewrite and reorganize the manuscript to make the main points more concisely and clearly - in the present form maybe only ~ 10 readers will be able to appreciate the study.

As mentioned in the previous response, according to the referee's suggestion we have attempted to re-write and re-organize the manuscript and we hope that the amended version brings the results in a more clear and precise manner.

2) reduce the drama in the intro and discussion - the point that existent model cannot exhibit the dynamics that the authors found is moot: the authors are using exactly the models they are criticising, just with the additional signal term. By the way, there were numerous numerical studies that used the same models with dynamic signal.

We thank the referee for this suggestion which we have taken into consideration when re-writing the manuscript. However, we would like to underline once again that our results do not only focus on analysis of previous models with temporally and spatially varying signals, but we additionally propose organization at criticality – before a saddle node of a pitchfork bifurcation (SubPB mechanism) – as a unique dynamical mechanism that enables transient but robust polarization features. The comparison with the remaining polarity models (LEGI, Turing-like and Wave-pinning) presented in Figs. 3-4 clearly demonstrates that the SubPB mechanism

uniquely allows to subsequently respond to transient signals from same direction, and is most efficient in re-polarization when signal direction is changed over time.

3) Explain clearly what is the novelty compared to your own eLife paper - it seems to me that all the main points were already made there.

In the amended version of the manuscript we emphasize that the current work proposes a conceptual framework to study cell polarization in a time-dependent form, as a non-autonomous process. In particular, we describe how the transient dynamics can be characterized by using a combination of bifurcation analysis, construction of quasi-potential landscape and local perturbation analysis. To the best of our knowledge, this is one of the first attempts to provide a framework for the analysis of transients and the non-autonomous nature of cellular models, a problem that has been relatively sparsely visited in the literature, since the characterization of possible dynamical behavior in these cases lies beyond simple numerical simulations. We therefore strongly believe that the proposed framework would serve as a basis to tackle the problems of transient dynamics, and to develop more sophisticated mathematical frameworks for analysis of non-autonomous cellular dynamics in the future.

Reviewer #3: Re: Review of Non-asymptotic transients away from steady states determine cellular responsiveness to dynamic spatial-temporal signals
----

The paper argues for a paradigm shift when considering models for cellular polarity. Cell polarization is crucial for cells to establish a front and back, and to organize cell cytoskeletal processes to facilitate cell migration. Understanding the biochemical networks establishing polarity is crucial to understanding cell migration. Polarization models most commonly are formulated as reaction-diffusion equations. Two main classes exist: (1) Turing based mechanisms; and (2) wave-pinning mechanisms.

The authors of this study argue that none of the existing models can recapitulate both (1) maintenance of a polarized state longer than the signal duration, and (2) retain responsiveness to novel signals. The authors encourage modellers in the field to embrace non-autonomous and non-asymptotic signalling dynamics as a solution. In particular, the authors demonstrate their argument with their recently developed SubPB dynamics. While I don't fully agree with the authors assessment in this regards (see some references suggesting otherwise below). I find that the authors point that we should consider non-autonomous dynamics an important one, and the referenced papers do essentially consider changes in time.

In my opinion the paper addresses an important question i.e.\ how to develop signalling networks that have all features observed in experiments. Further, in my opinion the paper argues for an important often neglected piece, that these signalling networks must respond to spatio-temporal variations. Frequently this means that transients and non-asymptotic dynamics are crucial for us to understand signalling networks.

The paper is generally well written, albeit confusing at times (see specific comments below). The major challenge for me was to understand what the SubPB model is, how it is defined, how it is different to Turing and wave-pinning models. Having worked on these models before, I was excited to understand how the authors addressed the above mentioned challenges, and tried for significant time to understand how the SubPB model works. However, I did not succeed. This is amplified by Figures that don't fully explain what is shown i.e. (conditions to replicate the figures, and sometimes what equations are used). Given this, I currently don't think that this paper is reproducible (code is not available, as far as I can tell). Having said this, I believe that these things can be fixed, and would encourage the authors to carefully revise their manuscript, with readability and reproducibility in mind. In particular, I would encourage the authors to not refer any of the mathematical and modelling details to their recent eLife publication.

Once the paper is revised, I will support its publication in PLOS CB.

We thank the referee for his/hers insightful comments and constructive criticism that we have implemented in details in the amended version of the manuscript. We address the specific comments as follows:

We are very grateful to the referee for pointing to us the unclarity in the descriptions in the previous version of the manuscript. In accordance with these comments, as well as taking the criticism of the other referees into account, we have re-written the manuscripts and re-structured the first two figures to better explain this point.

In particular, both the Wave-pinning at the SubPB mechanism rely on a subcritical pitchfork bifurcation for the realization of the symmetry-broken polar state. However, the Wave-pinning model is organized in a regime where in the absence of a signal, the non-polar and the polarized state coexist (region III in Figure 1B in the manuscript), whereas the SubPB model has parameter organization before the SN of the PB (region II in Figure 1B), or in a region where in absence of a signal only the non-polar state is stable.

Although the quasi-potential landscape calculated for the SubPB model has a single well in absence of a signal (in contrast to the Wave-pinning model that is characterized with three wells – one of the non-polar, homogeneous steady state and two corresponding to the two branches of the inhomogeneous steady state, the left- and right-polarized states), the closeness to the SN_{PB} generates, next to the single well, a shallow slope in the landscape. Thus, in a presence of a transient signal the landscape changes (one of a basic characteristics following the non-autonomous treatment of the system), and after the robust polarization and the signal removal, the system “falls” in the shallow slope part of the landscape. Due to the relatively flat structure, the system moves slowly, resulting in a transient memory of the polarized state. Dynamically, this state corresponds to a “ghost” of the SN bifurcation that has been lost upon signal removal. This state thus is metastable, and confers the flexibility to the system when responding to subsequent signals from same or changed direction. In contrast, in the Wave-pinning model, the system is maintained in the polarized state continuously (stable attractor, branch of the IHSS), and therefore some limitation in the responsiveness of the system arise, especially in responsiveness to transient signal from same direction.

In relation to the capability of the Wave-pinning model to respond to subsequent signals from different localization (re-polarization), what the referee raised in one of the following comments – we have included additional analysis in the current version of the manuscript. In particular, in order to demonstrate how the responsiveness in terms of re-polarization is affected when the polarized state results from a stable attractor (as in the Wave-pinning model) or metastable “ghost” state (as in the SubPB) model, we systematically characterized the amplitude and width of a signal necessary to repolarize the cell, as well as the time in which repolarization occurs for both models. The results show that re-polarization in the Wave-pinning model is possible only for signals with an amplitude $\sim 7 \times sd_{thresh}$. In contrast, the re-polarization in the SubPB mechanism can be achieved for reversed gradients with wide range of widths and amplitudes (Supplementary Fig. 3D in the amended version of the manuscript).

Major Comments
----

1) The major issue I encountered while reading this paper is that I am not clear what the SubPB mechanism is, and more importantly how it is different to the wave-pinning model. I did look at the referenced eLife article, but that didn't help. In my opinion, it would greatly improve the readability of this paper if the authors clearly define what the SubPB model is. Clarifying this may also address point (2) below.

In the amended version of the manuscript, we have addressed this point as described in the answer to the previous comment.

2) Equation (1). When s(t, theta) = 0, then given the authors choice of nonlinearities this becomes the wave-pinning model as introduced by Mori et al, featuring mass-conservation and stalling waves. The way the external perturbation is added here destroys the mass-conservation property. Other studies [3] and [4] below consider perturbations that leave the mass conservation property in tact, while [5] considered relaxing this assumption. Based on reference [5], I have the impression that relaxing the mass conservation property, substantially changes the dynamics of these models.

Having said all of this, I am unclear whether it's the authors intention to break mass conservation or not, and whether breaking mass conservation leads to different model behaviours. Once again, I think that I'm mainly unclear about the authors' assumptions, and what exactly the SubPB model is.

We thank the referee for this comment. Indeed, in order to preserve mass conservation, the signal has been subtracted from the v equation in all the analysis that is(was) presented in the paper. We have chosen to represent the signal s only in the u variable in the previous version of the manuscript as a means for simplified representation, but we understand that this might generate confusion in terms of the actual model implementation, and we have therefore amended it in the current version of the manuscript. Thus, the features observed are not a result of a broken mass-conservation, but rely on the presence of a “ghost” metastable state due to the organization before the SN_{PB} bifurcation, as outlined above, and in the current version of the manuscript.

3) Almost all of the Figures appear to be lacking the required information to reproduce the images i.e.\ numerical methods used, parameters, and initial conditions for the shown simulations.

We have previously included all of the parameters in the Materials and methods section, however for clarity, the amended version of the figure legends contain all the information necessary to reproduce the figures. This is also then complemented with the codes that have been released on github and are referenced in the manuscript.

4) The matched asymptotic analysis in Mori et al (2011) shows that the transition point from u_left to u_right is a function of the total mass in the system. A similar projection (as in this paper) was employed by Walther et al (2012). In this work, the transition point was still a function of the total mass, in reference to the asymptotic analysis by Mori et al (2011). Here the authors employ a projection that seemingly has a fixed transition point. How do the authors justify this?

Even in Turing systems without mass conservation the transition point in a ``mesa-pattern" is not centered in the domain. In fact matched asymptotic analysis by McKay and Kolokolnikov (2013) shows that its a function of the nonlinearities used.

Given this how good of an approximation is the used projection?

That the dynamical transition in the SubPB model can be described for fixed transition point comes due to the change in the formalism and the usage of the non-autonomous system description. In particular, under physiological conditions, the total amount of the system's constituents is conserved, but what changes are the environmental signals which are presented in a dynamic fashion to the cell. When considering a non-autonomous systems description (combined with the calculations of the bifurcation diagrams Fig. 1E, and quasi-potential landscapes Fig. 1D, G), it is straightforward to see that for fixed total concentration of the proteins, a dynamic signals induces changes in the potential landscape, such that certain solutions are stabilized/destabilized, thus naturally leading to the observed dynamical transitions from non-polar to polarized state, and transient maintenance of the polarized state after signal removal.

Studies looking at similar questions:

There are a number of studies both computational and theoretical that have examined similar questions recently, and not so recently, that in my opinion would merit mentioning.

1) Polarity and mixed-mode oscillations may underlie different patterns of cellular migration; Plazen et al (2023) in Scientific Reports, appears to address a similar question. In particular, the authors were motivated to modify and extend existing polarity models by experimental data showing cell polarization reorientation.

2) Deterministic Versus Stochastic Cell Polarisation Through Wave-Pinning (2012). A study that uses a similar projection, as considered in the present paper.

3) Cell Repolarization: A Bifurcation Study of Spatio-Temporal Perturbations of Polar Cells (2022) BMB. A study which appears to demonstrate that the wave-pinning models to retain sensitivity to subsequent signals, and re-orientation of the polarized states is possible. While this study is autonomous, it seems it could be re-interpreted in a non-autonomous setting.

4) Dynamics of localized unimodal patterns in reaction-diffusion systems for cell polarization by extracellular signaling (2018) SIADS. This paper considers the same setup as this study. A wave-pinning model on a periodic domain, with signalling inputs. This paper also suggests that existing wave-pinning models can respond to subsequent signals.

5) A Model for Cell Polarization Without Mass Conservation (2017) SIADS. Considers cell polarization models without mass-conservation. These models appear to be quite different in terms of the featured dynamics.

We are very grateful to the referee for pointing to us these studies that we have clearly missed. In the current version of the manuscript we discuss the relevant studies, and furthermore, we use the results of Ref. 3 as a basis to perform a more systematic parameter study and characterize the re-polarization features of the wave-pinning and the SubPB mechanism (shown in Supplementary Fig. 3D now). We would like to note that our study is limited to models with mass-conservation, as typical for signaling networks, and therefore we decided to restrain ourselves from discussing polarization models without mass conservation in the current manuscript.

Minor Comments

- In Figure 1A: The projected system appears to have non-periodic boundary conditions. This is of course fine, but the authors should mention why that is, and how to map the periodic to non-periodic domains. This is something that confuses many.

In order to calculate the quasi-potential landscapes, we made an approximation where the cell is considered to be constituted by 2 bins, a left and a right bin, that can exchange the reacting species thereby mimicking diffusion. Although a simplified geometry is considered, this model exhibits equivalent dynamical transitions as the full RD model (one dimensional domain of length $L=2\pi R$ with periodic boundary conditions), as demonstrated analytically (Materials and methods), and numerically (Fig. 1B).

- Figure 1F/G: Which model is this plot for? The projection or the full RDE?

The quasi-potential landscapes and the analysis of the transient dynamics of the system has been performed on the one-dimensional model projection (Eqs. (3) in the current version of the manuscript). We have furthermore specified which model is used for which analysis at the beginning of the results section after the model presentation, as well as in the figure legends.

- Supp Figure 3: Why is the SubPB mechanism not shown here?

The SubPB mechanism was not shown here as this data is/was given in Figure 1. In the amended version of the paper, we have re-structured the manuscript and discuss the wave-pinning model already in Figure 1B,C.

- Line 81: Which one-dimensional model are you referring to? The projection or the PDE?

As a one-dimensional model we always refer to the projection model, and we have specified this throughout the manuscript in the current version.

- Line 239: What characteristics?

Under characteristic we refer here to the realization of the IHSS. As discussed in the manuscript, the IHSS is a symmetry-broken steady state – a combination of a high activity at the cell left and low on the cell right or the other way around, and these right- and left-polarized solutions correspond to the two IHSS branches depicted in solid red lines in Figure 1B in the manuscript. Thus, when the system is stimulated with two spatially opposed signals with different amplitudes, the direction of the higher signal amplitude will robustly induce a transition to the corresponding IHSS branch. This feature is equivalent in the SubPB and in the wave-pinning model, as the polarization in both models relies on an IHSS.

- Line 315: The sentence seems incomplete.

We have corrected it.

- Line 330: Again I don't understand what the difference between wave-pinning and SubPB is. Later on Line 373 the authors state a c_total for the SubPB model, but I don't think it conserves mass. Second the wave-pinning models are only described by equation (1) if s = 0.

As noted in the responses to the previous questions, we have amended both the description in terms of the model to explicitly address the mass conservation, and specified in Figure 1B which regime organization corresponds to the Wave-pinning or the SubPB mechanism. We hope that together with the changes in the text and re-organization of the figure, this point is made clear in the current manuscript version.

- Line 334: The vectors should not be inline the text. Maybe use the transpose to make this look better.

We have amended this.

- Line 416: For the simulations the authors use only 20 discretization points. That seems really small. That gives a spatial step size of larger than 1/2, and since the global error of a central difference scales like O(h^2) a global error of larger than a 1/4. For a RDE with sharp transitions, I would expect that such a coarse domain discretization would not be able to accurately describe the evolution of the sharp interface.

In order to integrate the RD system, we used *method of lines* (described in details in the section Model implementation in Materials and methods in the amended text, and also in response to referee 1) to discretize the continuous RD equations. Numerical stability condition at every grid point of this finite difference method according to the von Neumann stability analysis is: $\frac{D\Delta t}{h^2} \leq \frac{1}{2}$, where D is the diffusion constant, Δt is the integration time step and h is the spatial grid step. Although the h value for the simulation was $\approx \frac{1}{2}$, a sufficiently low Δt value enables to achieve numerical stability. The ODE system that we obtained from the PDE (described in details in Materials and methods) was integrated using *solve_ivp* ODE integrator in *python*. In order to ensure numerical stability of the solutions, we used explicit Runge-Kutta method of order 5(4) with adaptive time step control. Truncation error of the integrator of the order $\sim O(\Delta t^6)$ was sufficient to capture sharp transitions.

- Line 503: The last and first name of the reference are flipped.

It is corrected.
